# A Study of Quantum Confinement Effects in Ultrathin NiO Films Performed by Experiment and Theory

**DOI:** 10.3390/ma11060949

**Published:** 2018-06-04

**Authors:** Christos S. Garoufalis, Alexandros Barnasas, Alkeos Stamatelatos, Vagelis Karoutsos, Spyridon Grammatikopoulos, Panagiotis Poulopoulos, Sotirios Baskoutas

**Affiliations:** 1 Department of Materials Science, University of Patras, 26504 Patras, Greece; garoufal@upatras.gr (C.S.G.); mparnalex@gmail.com (A.B.); alkstamatelatos@gmail.com (A.S.); vkar@upatras.gr (V.K.); poulop@upatras.gr (P.P.); 2 Department of Mechanical Engineering, Technological Educational Institute (T.E.I.) of Western Greece, M. Alexandrou 1, 26504 Patras, Greece; spiridongramma@upatras.gr

**Keywords:** thin films, semiconductor oxides, grain growth, optical properties, quantum confinement, potential morphing method

## Abstract

Ultrathin NiO films in the thickness range between 1 and 27 nm have been deposited on high-quality quartz substrates by direct magnetron sputtering under a rough vacuum with a base pressure of 2 × 10^−2^ mbar. The sputtering target was metallic Ni; however, due to the rough vacuum a precursor material was grown in which most of Ni was already oxidized. Subsequent short annealing at temperatures of about 600 °C in a furnace in air resulted in NiO with high crystallinity quality, as atomic force microscopy revealed. The images of surface morphology showed that the NiO films were continuous and follow a normal grain growth mode. UV-Vis light absorption spectroscopy experiments have revealed a blue shift of the direct band gap of NiO. The band gap was determined either by Tauc plots (onset) or by the derivative method (highest rate of absorbance increase just after the onset). The experimental results are interpreted as evidences of quantum confinement effects. Theoretical calculations based on Hartree Fock approximation as applied for an electron-hole system, in the framework of effective mass approximation were carried out. The agreement between theory and experiment supports the quantum confinement interpretation.

## 1. Introduction

NiO is a wide direct band gap semiconductor with an energy band gap *E_g_* close to 4 eV [1]. NiO is placed in the center of scientific attention for the realization of various applications, such as gas sensing [2,3], nonvolatile storage media [4], battery cathodes [5], and transparent UV detectors in the field of optoelectronics [6]. Moreover, it can be used in photovoltaics as a transparent oxide semiconductor [7,8] and in the formation of modern pn junctions, such as NiO/ZnO ones [9,10]. Among the most important properties of a nanoscaled semiconductor for optoelectronics is the controllable band gap tuning [11,12]. This tuning is achievable through the strong band gap dependence on the particle size or film thickness of the semiconductors and, therefore, it can be tailored by reducing the semiconductor dimensionality.

There are many studies dealing with the *E_g_* determination of NiO thin films and nanoparticles. In most of them, *E_g_* is found to be close to the bulk value, while there are also reports which indicate a “red” shift (towards lower energies) of *E_g_* (see e.g., [13,14,15,16]). However, due to the very small Bohr radius of NiO, it is difficult to find studies where a “blue” shift systematically develops with reduced dimensionality and investigated by experiment and theory. Some preliminary results were published earlier [17]. In the present contribution, we present a complete experimental study which is also supported and complemented by theoretical calculation based on the potential morphing method (PMM). We grow ultrathin NiO thin films with an alternative method which starts from the growth of a precursor NiO material in a rough vacuum followed by a short annealing time at 600 °C [18]. At such elevated temperatures NiO film crystallinity improves [19]. The growth habits of our NiO films with thicknesses ranging from 1 to 27 nm are briefly presented and discussed. Subsequently, our focus is turned on their optical absorption properties, which are found to exhibit a smooth increase of the *E_g_* position as the film thickness decreases. The maximum value of the blue shift observed in our experiments is around 0.4 eV and it corresponds to our thinnest (1 nm) NiO sample. The observed trends remain the same regardless of the adopted method of study (i.e., Tauc plots [20] or derivative method [12]). These two different approaches are compared and discussed in a comprehensive way.

The results are compared to theoretical calculations based on a two-particle (electron and hole) Hartree Fock (HF) approximation [21,22,23], which is solved in the framework of effective mass approximation (EMA). This choice of method is dictated not only by the fact that the size of the systems under consideration is by far too large for more robust and advanced approximation (e.g., DFT), but also because a detailed microscopic treatment exhibits difficulties (due to passivation issues) in capturing the quantum confinement effect. For example, it is well known that the (111) surface of NiO can adopt several types of reconstruction such as the so-called octopolar, 2 × 2 − α, and Rt3, which, in a microscopic theoretical treatment, must be explicitly considered. On top of this, in such a treatment, the passivation of the surface has to be done atom by atom and it might have a significant effect on the electronic properties (and the calculated gap) of the material. This becomes evident in the thorough and extensive DFT + U investigation of (111) NiO surface by Li et al. [24], which revealed that the calculations on three slabs with different thicknesses (1.43, 1.92, and 2.41 nm) produced the same gap ∼1.19 eV which, by the way, is also smaller than the calculated bulk gap (2.43 eV). As a result, we resort to the traditional and well behaved combination of HF and EMA, which has a proven record of successful treatments of such problems. The solution of the relevant equations is achieved numerically with the help of the potential morphing method (PMM) [25]. It should be noted that NiO is a natural p-type semiconductor due to deviations from stoichiometry [26]. Taking this into consideration, as well as the fact that the effective mass of doped semiconductors increases [27], we perform suitable calculations in order to study the influence of this effect on the “blue’ shift of *E_g_*. Our theoretical results for the dependence of the direct band gap of NiO on the film thickness are in good agreement with the experimental values. This agreement verifies that the experimentally-observed shifts can be safely attributed to quantum confinement effects.

## 2. Materials and Methods

### 2.1. Experimental Details

Ultrathin NiO films with thicknesses in the range of 1–27 nm were deposited on quartz glass by direct current (DC) magnetron sputtering. The Ni target was 99.95% pure and was purchased by Alfa Aesar ((Karlsruhe, Germany). The deposition temperature was the room temperature. We used a simple table sputter coater by Balzers (Balzers Union model SCD040, Liectenstein). Therefore, the base pressure of the vacuum chamber was 2 × 10^−2^ mbar. The total pressure (including Ar partial pressure) during deposition was about 5 × 10^−2^ mbar.

Film thickness evaluation was performed with the help of AFM, Multimode, (Bruker, Santa Barbara, CA, USA) images of the profile of a narrow scratch done intentionally on the film surface [28]. The AFM was a multimode microscope with a Nanoscope IIIa controller and a 120 μm × 120 μm magnet-free scanner (model AS-130VMF) developed by Bruker (Santa Barbara, CA, USA). The microscope was operated in the non-contact (tapping) mode [29].

As we have recently shown for Au–Ni–O films [18], under such growth conditions in a rough vacuum, a precursor thin film material is formed. This may contain a major part of NiO and to a less extent metallic Ni. In this work, selected samples were measured via energy dispersive X-ray spectroscopy (EDS) with our scanning electron microscope (SEM) a Zeiss EVO MA 10 model (ZEISS, Oberkochen, Germany). It was found that the ratio of Ni and O atoms within the experimental error was almost 1, as it should be in NiO. In order to obtain high-quality nanocrystalline samples [19], and strain annihilation, the films were subsequently annealed for 20 min at 600 °C in air. The films’ microstructure was then monitored by AFM experiments.

Finally, the ultraviolet UV–Vis spectra were recorded at room temperature in the transmission geometry with the help of a Perkin Elmer Λ-35 UV–Vis spectrometer (PerkinElmer, Akron Ohio, USA) at the wavelength range 200–1100 nm.

### 2.2. Theory

In the effective mass approximation the Hamiltonian for the electron hole system can be written as [21,22,23]:

(1) $$H=-\frac{\hbar^{2}}{2m_{e}^{*}}\nabla_{e}^{2}-\frac{\hbar^{2}}{2m_{h}^{*}}\nabla_{h}^{2}+V_{0}^{e}\left(r_{e}\right)+V_{0}^{h}\left(r_{h}\right)-\frac{e^{2}}{\varepsilon }\frac{1}{r_{eh}}$$

where $m_{e}^{*}$ $\left(m_{h}^{*}\right)$ is the effective electron (hole) band mass, ε is the size dependent effective dielectric constant [30], *r_eh_* is the electron-hole distance and $V_{0}^{e\left(h\right)}$ is the confinement potential of electron (hole). The Hartree-Fock formulation of two particles (electron and one hole) results in the following coupled equations:

(2) $$\left[\frac{p_{i}^{2}}{2m_{i}^{*}}+U_{i}\left(r_{i}\right)\right]\Phi_{i}\left(r_{i}\right)={\tilde{E}}_{i}\Phi_{i}\left(r_{i}\right)\;i=e,h$$

The self–consistent effective field *U_i_*(*r_i_*) that acts on the particles includes the interaction with the confining potential as well as the Coulomb and exchange interaction between the two particles [21,22,23]. Once the numerical solution has been achieved [21,22,23], the total energy of the exciton, is calculated as $E\left(X\right)={\tilde{E}}_{e}+{\tilde{E}}_{h}$, while the corresponding effective band gap is obtained by adding the exciton energy to the band gap of the bulk material:

(3) $$E_{g}^{eff}\left(X\right)=E_{g}+E\left(X\right)$$

A more detailed description of the adopted methodology (including technical details of the calculations) can be found elsewhere [21,22,23]. In the specific implementation, the shape of the confining potential, was assumed to be *V*_0_ outside the film and zero inside. The value of *V*_0_ is empirically determined as described in previous studies [22]:

(4) $$V_{0}^{e\left(h\right)}=\{\begin{array}{cc} 0 & \left|z\right|<L/2\\ V_{0} & \left|z\right|\geq L/2 \end{array}$$

## 3. Results 

### 3.1. Grain Growth

**Figure 1:**
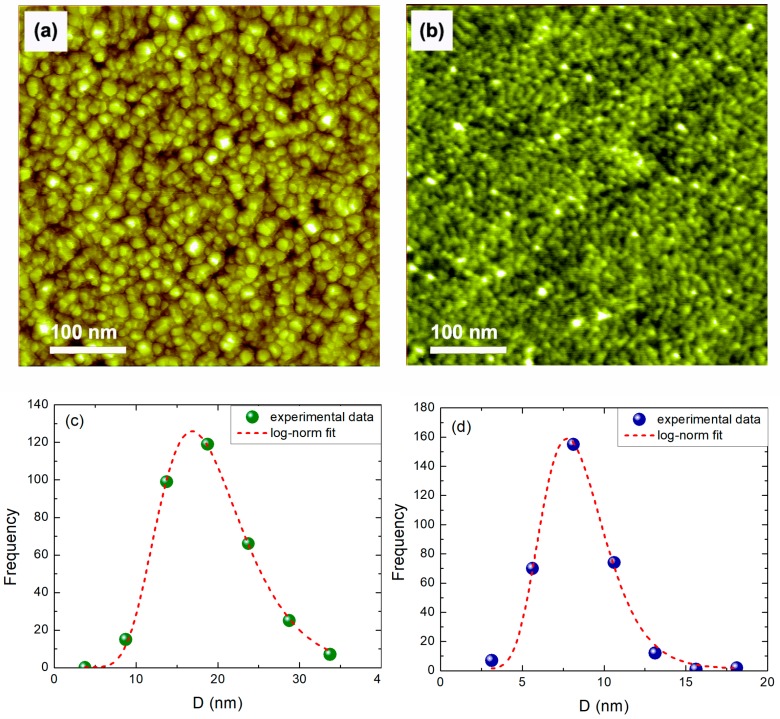
AFM images of (**a**) 13.5 nm and (**b**) 4 nm thick NiO films. The image size is 500 × 500 nm^2^. Grain diameter *D* size distributions for the (**c**) 13.5 nm and (**d**) 4 nm thick NiO films.

Atomic force microscopy (AFM, Multimode, Bruker, Santa Barbara, CA, USA) experiments have been performed for all films with thickness *t* ranging between 4 and 27 nm. The resolution of the AFM was not adequate enough to probe thinner films. In Figure 1a one may see, as a typical example, the AFM image from the surface of a 13.5 nm thick NiO film. It is noteworthy that our AFM image unambiguously shows a facetted morphology of NiO and nanocrystals with good symmetric shape. This is a strong evidence of sample crystallinity. A similar AFM image from a 4 nm thick NiO film is also presented in Figure 1b. Both films are smooth and continuous. The surface roughness is relatively small and the nanocrystallites (grains) seem to be quite homogeneous in terms of size. In Figure 1c,d we plot the grain diameter *D* size distribution of the two films of Figure 1a,b, respectively. One may observe that both distributions are well-described by the log-norm function [31]. The average values of *D* are 16.8 nm and 7.7 nm for the 13.5 and 4 nm thick films. The error in the determination of the grain size increases for thinner films as *D* approaches the tip radius of 7 nm. The full-width at half-maximum (FWHM) is 12.1 nm for the first film and 4.7 nm for the second. Therefore, both distributions are relatively narrow.

**Figure 2:**
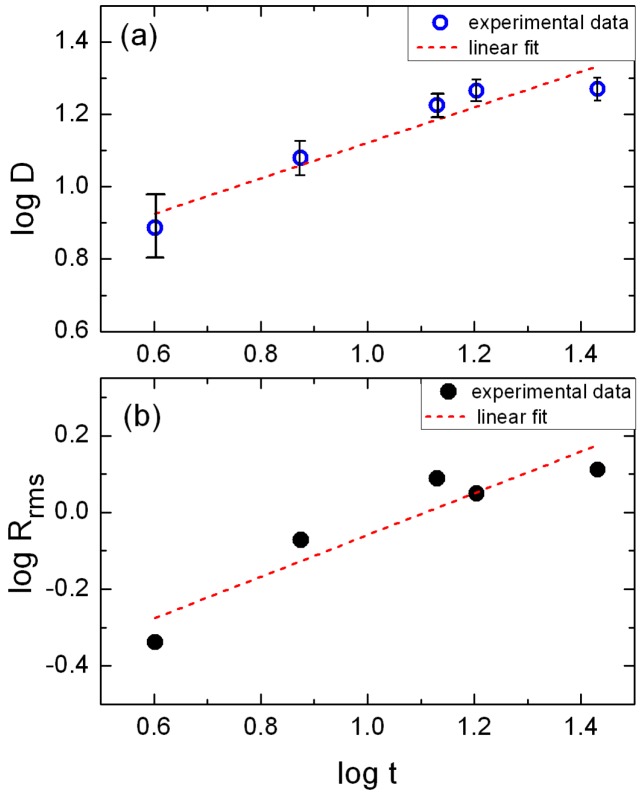
(**a**) Grain diameter D as a function of film thickness *t* in a log-log plot. The slope of the linear fit has a value consistent with normal grain growth mode. The error bar increases with decreasing film thickness due to the finite tip radius of 7 nm. (**b**) root-mean-square roughness values as a function of film thickness for the same five NiO samples the thickness of which is in the range of 4–27 nm.

In Figure 2a we attempted to plot the log-log plot for the grain diameter as a function of film thickness. This type of plots may provide some indirect information on the grain growth mode of thin films [32,33,34,35]. The slope of the line after linear regression is 0.49 ± 0.09. This value is very close to the theoretical and computational values for normal grain growth mode [36,37]. One has to recall that our films’ images were recorded after short annealing at a relatively high temperature. This temperature of 873 K, however, is much smaller than the melting point of NiO which is about 2228 K. Therefore, we are far below the recrystallization temperature. NiO grains in our films may become larger after short annealing than in the as-deposited state, but they do not grow the one in the expense of the other; the latter is typical at the recrystallization temperature and above. Thus, the normal grain growth feature of homogeneous monomodal grain-size distribution is maintained. This is confirmed both, by the AFM images of Figure 1 and Figure 2a.

Average root-mean-square roughness values, *R*_rms_, as a function of film thickness for the five NiO samples studied by AFM is plotted in Figure 2b. According to the log-log plot of this figure the roughness increases with thickness following a power law with the form *R*_rms_ ~ *t*^b^, where *b* is the slope of the best fitted line which is found to be *b* = 0.54 ± 0.12. The aforementioned power law has been predicted by many theoretical models for polycrystalline thin films [38,39], where the exponent *b* is in the range of 0.3–0.9 (see, for example, [34]). Surface roughness measurements made on the same system, i.e., NiO films, grown on Si wafers at 200 °C by atomic layer deposition, with a similar thickness as our films (6–29 nm), resulted in a *b* = 0.94 [40]. We notice that there is a significant deviation with the exponent value obtained by our measurements. However, we have to bear in mind that the roughness data are nothing more but statistical values about the surface height information and these value depend on many factors, such as the deposition technique, film substrate, substrate temperature, as well as which surface area is studied [34].

### 3.2. Optical Properties

**Figure 3:**
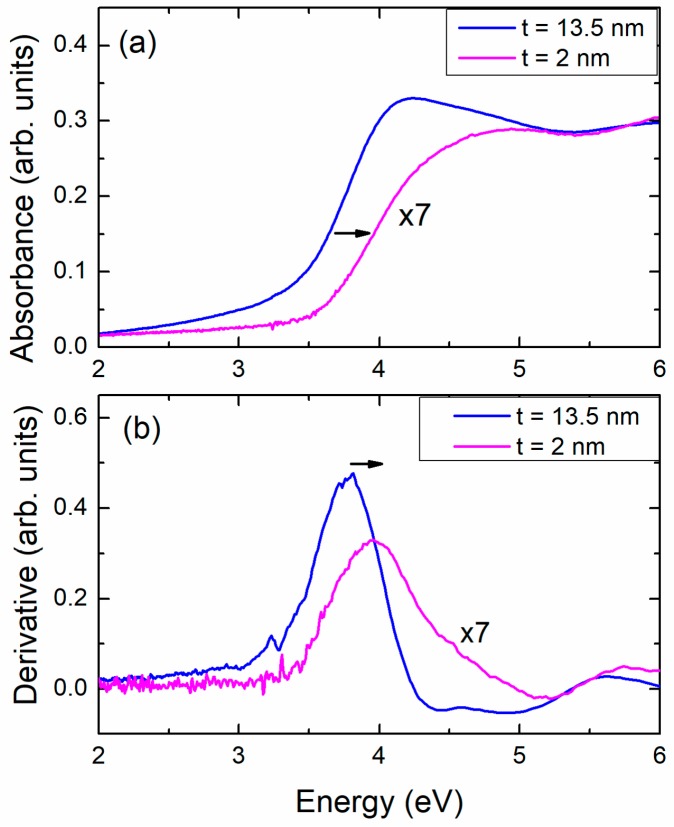
(**a**) Absorbance spectra for two NiO films. The film thickness is indicated. The absorbance of the thinner film has been multiplied by 7 for better clarity. One may see a “blue” shift of the energy band gap as determined by the first derivative of the spectra, see (**b**).

In Figure 3a we plot the optical density (or absorbance *A* = −log*T*, *T* is the transmittance [20]) spectra, for two NiO films. In Figure 3b the first derivative of *A* is shown. The first derivative maximum marks the point where one encounters the highest rate of the absorbance increase just after the onset of *E_g_*. The arrows indicate a clear “blue” shift of the spectra and of the derivative maxima as the film thickness decreases. Although there is a debate on the use of either the derivative, or the more commonly used Tauc plots, for the study of the blue shift of the energy gap and the features of the absorbance spectra, the current NiO samples give us an excellent opportunity to go into some more details since the spectra are almost free of noise.

**Figure 4:**
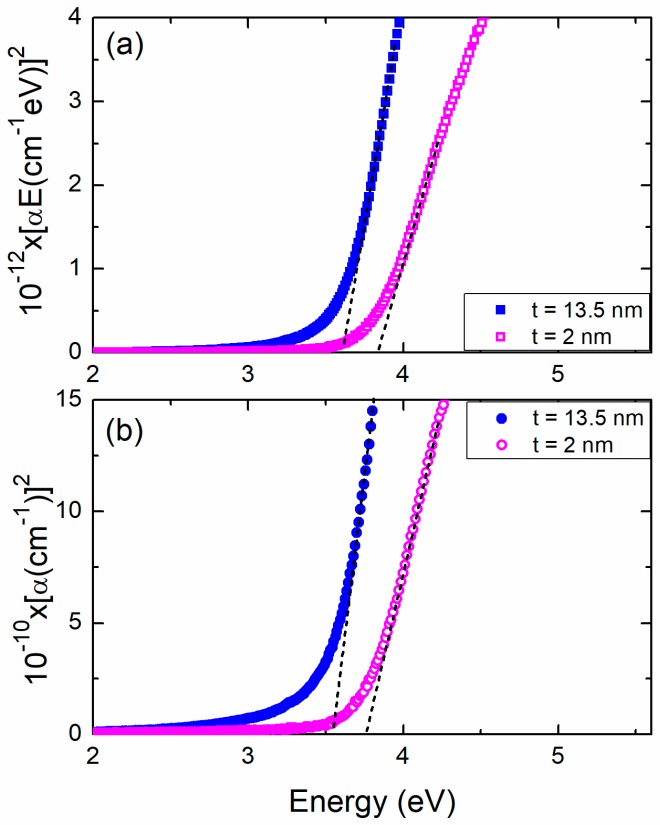
Tauc plots for two NiO films. (**a**) (α*E*)^2^ as a function of photon energy *E* and (**b**) (α)^2^ as a function of E. One can clearly observe a “blue” shift of *E_g_* determined with high precision by the extrapolation of a rather large linear part of the plots. The film thickness is indicated.

Therefore, for the two aforementioned films (*t* = 13.5 nm and *t* = 2 nm) we present in Figure 4, two commonly adopted approaches of a Tauc plot for a semiconductor with direct *E_g_* and dipole-dipole allowed transitions. In particular, Figure 4a shows the magnitude of (α*E*)^2^ as a function of photon energy *E*, e.g., [13,14,15,16], while Figure 4b depicts the variation of α^2^ as a function of *E* [20] (α is the absorption coefficient). One may observe that there is a large almost linear part of the curves near the onset of *E_g_*. The intercept with the energy axis is *E_g_*. In most cases in literature, one deals with relatively thick films. Rayleigh scattering at the grain boundaries, defects or impurities add a parabolic slope to the whole spectrum of the absorbance [20]. This effect limits the linearity of the Tauc plots. Therefore, they exhibit a degree of uncertainty depending on how straight a Tauc curve is. On the contrary, in most cases, the derivative method is found to be more accurate since the maximum of *dA/dE* can be safely determined. In our case the quality of the data, which reflects the quality of the films, makes the linear part of Tauc plots significant, see Figure 4, and increases the accuracy of the determined onset of *E_g_*.

**Figure 5:**
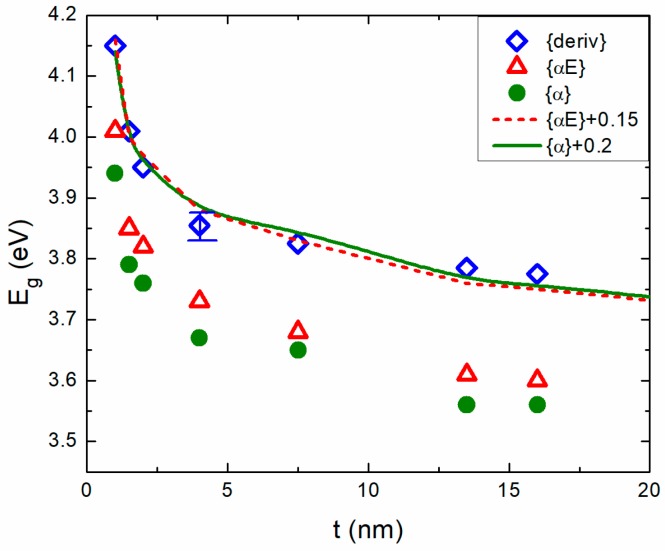
All features of absorbance spectra such as the onset of *E_g_* determined by the Tauc plots and the maximum of absorbance after the onset determined by the derivative maximum, coincide.

The results of the derivative method and the two Tauc plots are collected and presented in a comparative manner in Figure 5. Moreover, with the lines, we present the sum of the onset of *E_g_* determined by the Tauc plots and a vertical shift constant. We see that the two lines coincide, within the experimental accuracy, with the derivative data. Therefore, it is safe to conclude that all these values of the optical-properties (i.e., *E_g_* onset and maximum absorbance increase rate) exhibit the same behavior (i.e., they increase as the film thickness decreases). Indeed, this becomes even more clear if one goes to the thinner films where quantum confinement effects are enhanced since the film thickness becomes comparable to the Bohr radius of the excitons.

**Figure 6:**
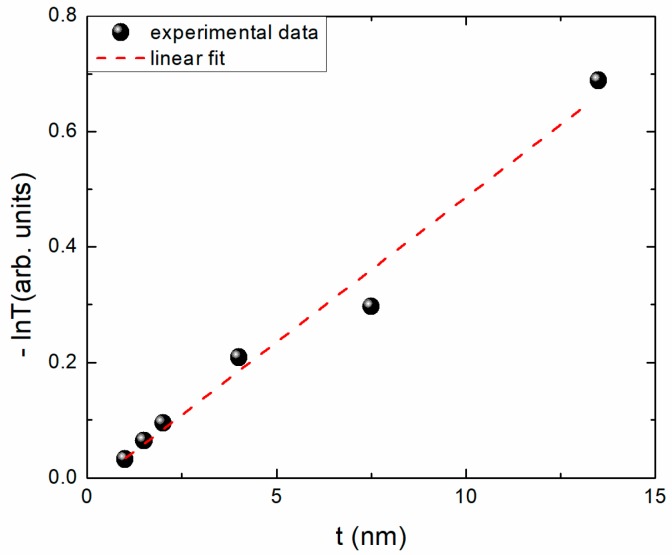
(−ln*T*) as a function of film thickness *t*. The slope of the linear fit is equal to the absorption coefficient. The transmittance T data have been recorded at 5 eV (250 nm).

For completeness sake, in Figure 6 we present the absorption coefficient determination by the slope of a (–ln*T*) as a function of film thickness t diagram. We select a photon energy of 5 eV (wavelength λ of 250 nm), because this energy position in all spectra, even the “blue” shifted ones, is after the edge jump. Our value of α at 250 nm is equal to 5 × 10^5^ cm^−1^, which is in fair agreement with previous reports on high-quality films [1].

## 4. Discussion and Conclusions

Any calculation based on the effective mass approximation is heavily dependent on the supplied values of electron and hole effective masses (m_e_*, m_h_*). However, for the case of NiO, the values found in the literature appear to be extremely diverse. It is worth noting that in many cases, even the reported trends, are not consistent with each other [27,41,42,43,44]. To some extent this diversity might be related to changes in the conduction properties (i.e., p-type semiconductor, etc.) which are affected by variations in stoichiometry and, in turn, might also modify the value of the masses. As a result, we performed several benchmark calculations following the diverse literature suggestions, until we finally adopted the conclusion of Irwin et al. [27] who propose that the electron and hole exhibit similar masses which range from 0.5 to 1.0*m*_0_ (i.e., m_e_* ≈ m_h_* = 0.5 − 1.0*m*_0_). Furthermore, in order to include the dependence of the dielectric constant on the film thickness we have chosen to incorporate into our model the size-dependent dielectric function of Hanken [45] with the following parameters: *ε*_∞_ = 5.7, *ε*_0_ = 12.4 [46] and *ω*_LO_ = 64.47 meV [47].

**Figure 7:**
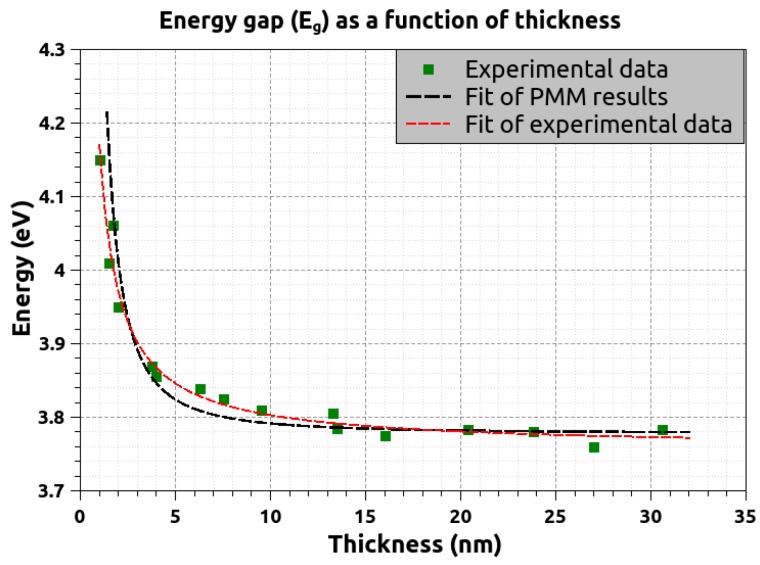
Direct band gap energy *E_g_* of ultrathin NiO films as a function of film thickness *t* by experiment and theory. Some experimental data from Ref. [17] have been also included. The black dashed line is derived by fitting the calculated values of *E_g_* on *E_g_* = *a* + *b*/*t*^c^ (*a*, *b*, and *c* are fitting parameters and t is the film thickness) while the red dashed line is a fit of the experimental data on the same function.

The results for m_e_* ≈ m_h_* = 0.8*m*_0_ are presented in Figure 7 along with experimental data. It is evident that the agreement between theory and experiment is quite good. It is worth noting that when the PMM values (produced by all combinations of masses) are fitted on *E_g_* = *a* + *b*/*t*^c^ formula, the *c* exponent ranges from 1.5 to 1.7, while for the experimental data the c parameter is closer to 1.0 (in simple effective mass theory, *c* is expected to be 2.0). In conclusion, the apparent similarity of the two curves presented in Figure 7 is highly suggestive that the observed shifts are a manifestation of the quantum confinement effect.

In this work we have studied microstructural features, i.e., grain size and surface roughness as a function of thickness up to 27 nm for ultrathin NiO films grown by direct current magnetron sputtering in rough vacuum and annealed shortly at 600 °C. Moreover, we have recorded the absorbance spectra of these films. We determine the onset of the energy band gap *E_g_* by using Tauc plots and the maximum rate of the absorbance increase after *E_g_* by the derivative method. Both features show a smooth increase, a “blue” shift, with decreasing NiO film thickness down to 1 nm. The experiment is well described by effective mass Hartree Fock calculations revealing that the blue shift is due to quantum confinement.
